# Racial Disparity in Stroke Awareness in the US: An Analysis of the 2014 National Health Interview Survey

**DOI:** 10.4172/2155-9562.1000365

**Published:** 2016-04-07

**Authors:** Nwakile Ojike, Joe Ravenell, Azizi Seixas, Alina Masters-Israilov, April Rogers, Girardin Jean-Louis, Gbenga Ogedegbe, Samy I McFarlane

**Affiliations:** 1 Center for Healthful Behavior Change (CHBC), Division of Health and Behavior, Department of Population Health, New York University Medical Center, New York, USA; 2 The Saul R. Korey, Department of Neurology, Albert Einstein College of Medicine, Bronx, NY, USA; 3 Division of Endocrinology, department of Medicine, SUNY-Downstate, Brooklyn, NY, 11203, USA

**Keywords:** Stroke, Race, Gender, Disparity, Hypertension, Awareness

## Abstract

**Background/Aims:**

Stroke is a leading cause of premature death and disability, and increasing the proportion of individuals who are aware of stroke symptoms is a target objective of the Healthy people 2020 project.

**Methods:**

We used data from the 2014 Supplement of the National Health Interview Survey (NHIS) to assess the prevalence of stroke symptom knowledge and awareness. We also tested, using a logistic regression model, the hypothesis that individuals who have knowledge of all 5 stroke symptoms will be have a greater likelihood to activate Emergency Medical Services (EMS) if a stroke is suspected.

**Results:**

From the 36,697 participants completing the survey 51% were female. In the entire sample, the age-adjusted awareness rate of stroke symptoms/calling 911 was 66.1%. Knowledge of the 5 stroke symptoms plus importance of calling 911 when a stroke is suspected was higher for females, Whites, and individuals with health insurance. Stroke awareness was lowest for Hispanics, Blacks, and survey participants from Western US region

**Conclusion:**

The findings allude to continuing differences in the knowledge of stroke symptoms across race/ethnic and other demographic groups. Further research will confirm the importance of increased health literacy for Stroke management and prevention in minority communities.

## Introduction

Stroke is a serious public health problem that accounts for 5% of all deaths in the US, and ranks No. 2 among all causes of death [1]. There are substantial racial, sex, and geographic disparities in stroke mortality, with higher rates in the South eastern United States, known as the stroke belt [2–4]. Low rates of stroke awareness and disease recognition among high-risk populations potentially limit acute stroke care and serve as a barrier to early and effective interventions [5].

Data indicates that only 51% of stroke patients arrive to the hospital in an ambulance thus hindering the prehospital care that could be provided to stroke victims [6]. This is quite concerning given the ample evidence that early intervention such as thrombolytic therapy is associated with reduced mortality, and higher rates of independent ambulation and discharge to home following stroke [7,8]. Increasing the proportion of individuals who are aware of stroke symptoms and the imperative of calling emergency medical services (911) is an objective of the Healthy people 2020 project [9].

The rationale of this study is to evaluate current race, sex, and regional differences in the rate of stroke symptom knowledge, as well as awareness of the importance of calling 9-1-1 on suspicion of stroke. We hypothesize that racial differences as well as sex differences exist in the rate of stroke awareness and activation of the emergency medical services that could potentially, at least-in-part account for the disparate mortality and morbidity rate among various populations. These differences could be readily addressable through public health educational campaigns targeting the low rate of awareness and high-risk populations.

## Methods

The study sample was derived from the 2014 NHIS Supplement data [10]. The NHIS is conducted annually by the National Center for Health Statistics (NCHS), Centers for Disease Control and Prevention (CDC). The procedures involved in the NHIS and details concerning its sample design can be found in “NHIS Survey Description–2014 Public Use Data Release” [11]. Briefly, the NHIS survey comprises a complex multistage area probability design that provides a representative sample of US households from all 50 states and the District of Columbia. A total of 36,697 individuals completed the survey corresponding to a response rate of 58.9%.

Participants provided answers to the questions concerning the symptoms of stroke-Which of the following would you say are the symptoms that someone may be having a stroke? “… Sudden numbness or weakness of face, arm, or leg, especially on one side” or “Sudden confusion or trouble speaking” or “sudden trouble seeing in one or both eyes “ or “Sudden trouble walking, dizziness, or loss of balance” or “severe headache with no known cause”. In addition, participants were asked to select the one action they would do first, from the following list of actions, if they thought that someone was having a stroke: take the person to the hospital, advise the person to call a doctor, call 911, call a spouse or family member, or other (do something else).

An individual was aware of stroke symptoms if they currently identified the 5 stroke symptoms, and selected rapid calling of 9-1-1 when a stroke occurs. Study demographics were outlined using descriptive statistics. Chi-squared test was used to describe the difference in proportion of stroke symptom awareness across study demographic factors. Differences between percentage point estimates were evaluated using 2-tailed significance tests at the 0.05 level. We calculated the age-adjusted prevalence rates using the 2010 USA standard population [12]. The application of observed age-specific rates to a standard age distribution diminishes any confounding from differences in crude rates in target populations that occur due to variations in the studied sample’s age distributions [13]. A logistic regression model, adjusted for risk variables, calculated the odds ratios (ORs) for calling 911 with awareness of five (5) stroke symptoms as specified above. To account for the multi-stage complex sampling design of the NHIS, study estimates are based on weighted data that was analyzed using SAS survey procedures (SAS Cary, NC).

## Results

Of the 36697 study participants 55% were women, 66.9% White, 11.9% Black, 5.6% Asian, 15.6% Hispanics, and 2.6% others. The age-adjusted rate of stroke awareness was 66.1%. Awareness of stroke symptoms was: 93.7% for sudden weakness of the face or limb, 92.8% for confused/troubled speech; 82.8% for trouble seeing, 90% for trouble walking/dizziness/loss of balance, 76.0% for sudden severe headache, and 95.3% for prompt activation of emergency medical response by dialling 9-1-1 (Table 1).

The least recognized individual stroke symptoms were sudden severe headaches (76.0%), and trouble seeing (82.8%). Correct awareness of the 5 stroke symptoms plus importance of calling emergency medical response was higher for females, Whites, and individuals with health insurance, and lowest for survey participants from Western US region (Table 2). Foreign-born Whites and Asians, had higher stroke symptom knowledge and awareness rates than their US born counterparts, while US born Hispanics had higher awareness rates. Awareness rates for foreign-born and US born Blacks was similar.

Correct identification of all 5-stroke symptoms was associated with a greater likelihood of calling 911, when compared with identification of fewer than five symptoms (Table 3).

## Discussion

We observed that the rate of stroke symptom awareness (including knowledge about calling 911 in the event of a stroke) was 66.1%. Knowledge of stroke symptoms varied by race/ethnicity, sex, region/location, but not by level of education or insurance coverage. Early recognition of stroke symptoms and calling 911 for rapid initiation of pre-hospital care has been universally included in various national and international stroke management guidelines [14,15]. Most recently,, 3 US centers utilizing mobile stroke units that are equipped with imaging and, lab facilities, as well as capability for early determination of stroke together with administration of thrombolytic therapy, these interventions showed a very high rate of early intervention and more than 50% of full recovery rate within 90 days post stroke [16].

Previous research using the Behavioral Risk Factor Surveillance System (BRFSS) survey, that was used in 13 states and the District of Columbia (DC), show stroke symptom awareness rates (including the priority of calling 911) of 38% and 17.2% [17,18]. Reports by the U.S. Department of Health and Human Services’ Healthy People 2020 project show a stroke symptom awareness rate of 51.2% in 2009 [19]. Compared with these early studies, our report of 66.1% might suggest a modest improvement in stroke symptom awareness. Furthermore, in contrast with the BRFSS, which is a state-based telephone survey that includes households with telephones in 12 states, the NHIS survey involves in-person interviews of individuals identified from geographically defined primary sampling units (PSU) covering the 50 States and the District of Columbia. The BRFSS questionnaire included in a discriminating question (“Do you think sudden chest pain or discomfort is a symptom of stroke?”) to assess the possibility that respondents could answer “yes” to all of the questions without thinking. Thus because the stroke knowledge section of the NHIS survey consists only of structured closed-ended questions, it is possible that our result overestimates participant knowledge and awareness of stroke.

Despite the increase in awareness of symptoms, the disparities in stroke symptoms persist across race/ethnic groups. This mirrors previous reports of continuing disparities in stroke mortality with declining overall stroke mortality rates [20]. Asians and Hispanics showed lower awareness rates of all 5 stroke symptoms, as compared to Whites and Blacks. While our study cannot provide exact reasons for the lower rates of stroke symptom awareness among Hispanics and Asians in the US, language barrier and associated low health literacy provide plausible explanations for our findings. For example, in a previous study conducted through the Health Interview Survey in California [21] involving over 48,000 adults, those with limited-English proficiency had nearly 4-times the rate of low health literacy levels compared English speaking group [21]. Interestingly in this study, Asians (Chinese, Koreans, and Vietnamese) had the lowest level of health literacy followed by Latino groups. The investigators concluded that individuals with both limited-English proficiency and low health literacy are at higher risk for poor health. However, limited-English proficiency may carry greater health risk than low health literacy. Health literacy is defined in Healthy People 2010 as the “the degree to which individuals have the capacity to obtain, process, and understand basic health information and services for appropriate health decisions” [22].

We observe that participants from the Southern United States had the highest level of awareness for stroke symptoms. This is despite higher prevalence of stroke in the region both historically [23], and in the current study. Gillum et al. showed that the excess risk for stroke occurrence in the South was independent of regional differences in prevalence of stroke risk factors especially for Whites [23]. Earlier studies demonstrated regional and racial differences in stroke mortality rates across the 11 states (Alabama, Arkansas, Georgia, Indiana, Kentucky, Louisiana, Mississippi, North Carolina, South Carolina, Tennessee, and Virginia) that make up the stroke belt [24–27]. The likelihood of calling 911 was higher for individuals who identified all 5 stroke symptoms compared to individuals who identified fewer than five symptoms. While other factors beyond the typical stroke symptoms could conceivably affect the recognition and early intervention in stroke population including mental capability and decision making, our data is limited by lack of such potential confounding that could possibly affect the awareness rate and hence the outcomes.

We show continuing disparities in awareness of stroke symptoms, and knowledge of the imperative of telephoning 911 when a stroke is suspected. It is expected that sustained public health efforts to improve stroke literacy, with an emphasis on men, nativity, Hispanics, Blacks, and Asian population, and provision of coverage to the still uninsured will help improve stroke management and prevention.

## Figures and Tables

**Table 1:** Age-adjusted rate of individuals who are aware of stroke. Symptoms/Signs and the importance of rapidly calling when a stroke occurs.

	No of Individuals	Sudden numbness or weakness of face, arm, or leg, especially on one side	Sudden confusion or trouble speaking	Sudden trouble seeing in one or both eyes	Sudden trouble walking, dizziness, or loss of balance	Sudden severe headache with no known cause	Would Call 9-1-1 Right Away if Someone was having a Stroke
ALL	36,697	93.7 (92.9–94.4)	92.8 (91.9–93.6)	82.8 (81.5–84.1)	90.0 (89.0–91.0)	76.0 (74.6–77.4)	95.3 (94.5–96.1)
Sex							
Male	16,398	93.0 (91.8–94.2)	91.9 (90.7–93.2)	81.9(80.1–83.7)	89.2(87.8–90.6)	73.9(71.8–75.9)	94.7 (93.4–95.9)
Female	20,299	94.3 (93.3–95.4)	93.6 (92.5–94.6)	83.7(81.9–85.5)	90.8(89.5–92.0)	77.9 (76.0–79.6)	95.8 (94.9–96.8)
Race/Ethnicity							
Whites	22779	96.3 (95.5–97.1)	96.0 (95.1–96.8)	86.6 (85.0–88.2)	92.9(91.8–94.0)	78.4 (76.5–80.2)	96.2(95.2–97.2)
Blacks	4896	92.9 (90.7–95.1)	92.1 (89.9–94.3)	80.3 (76.9–83.7)	89.3(86.7–91.9)	75.0 (71.3–78.7)	95.8(94.0–97.5)
Asians	2025	85.4 (81.4–89.6)	82.8 (78.3–87.4)	73.9 (68.6–79.2)	82.5(77.9–87.0)	69.1 (63.8–74.5)	91.2(87.5–94.9)
Hispanics	6053	85.5 (82.9–88.1)	82.8 (80.7–85.7)	72.4 (69.0–75.7)	80.7(77.7–83.7)	69.3 (65.7–72.8)	92.0(90.1–93.9)
Education							
<High School	5188	93.3 (92.4–94.2)	92.7 (91.7–93.7)	82.6 (81.0–84.1)	89.8 (88.6–91.0)	75.2 (73.5–76.9)	95.1 (94.2–96.0)
High School/Equiv	9196	93.5 (92.7–94.2)	93.1 (92.3–93.9)	83.6 (82.4–84.8)	90.3 (89.4–91.2)	76.5 (75.2–77.7)	95.3 (94.6–96.0)
Some College	11160	93.7 (93.0–94.3)	92.5 (91.8–93.1)	82.8 (82.0–83.7)	89.6 (88.8–90.4)	75.9 (74.6–77.1)	94.9 (94.1–95.7)
College/Grad degree	10612	94.0 (93.3–94.7)	92.9 (92.2–93.6)	82.2 (81.2–83.2)	90.5 (89.5–91.1)	76.3 (75.2–77.4)	95.7 (95.1–96.3)
Insurance Coverage							
Not Covered	4830	93.0 (91.8–94.1)	92.4 (91.3–93.5)	82.4 (80.6–84.1)	88.7 (87.4–90.0)	75.4 (73.7–77.2)	94.4 (93.4–95.5)
Covered	31674	93.8 (93.4–94.2)	92.8 (92.4–93.3)	82.8 (82.2–83.4)	90.2 (89.7–90.6)	76.1 (75.5–76.8)	95.4 (94.9–95.8)
Region							
Northeast	5919	93.4 (92.4–94.5)	93.3 (92.4–94.1)	84.0 (82.6–85.4)	88.9 (88.0–89.9)	77.1 (75.7–78.6)	94.5 (92.9–96.1)
Midwest	7809	94.9 (94.1–95.6)	94.0 (93.3–94.8)	83.6 (82.5–84.7)	91.2 (90.2–92.2)	75.1 (73.7–76.6)	96.4 (95.8–97.0)
South	12896	94.1 (93.5–94.8)	93.2 (92.5–93.9)	83.4 (82.4–84.4)	90.9 (90.1–91.6)	78.1 (77.1–79.1)	94.9 (94.1–95.7)
West	10073	91.8 (90.9–92.6)	90.4 (89.5–91.4)	80.0 (79.0–81.0)	88.1 (87.1–69.0)	72.7 (71.5–73.9)	95.7 (95.1–96.3)

**Table 2:** Age-adjusted rate of individuals who correctly identify all 5 stroke. Symptoms and would first call 911 in the event of a stroke.

	N	% (95% CL)	p
ALL	36658	66.1 (65.4–66.9)	
Sex			
Male	16374	63.6 (62.5–64.6)	
Female	20284	68.6 (67.5–69.7)	0.01
Race/Ethnicity			
**Whites**	22757	70.3 (69.4–71.2)	
US Born	18472	70.1 (68.9–71.1)	
Foreign Born	4247	71.6 (69.6–73.7)	
**Black**	4890	63.4 (61.2–65.6)	
US Born	3967	63.4 (61.0–65.8)	
Foreign Born	914	63.4 (59.7–67.2)	
**Asian**	2023	56.0 (53.2–58.9)	
US Born	944	55.2 (52.0–58.3)	
Foreign Born	361	60.3 (53.8–66.7)	
**Hispanic**	6045	54.3 (52.5–56.1)	
US Born	4851	54.7 (52.6–56.7)	
Foreign Born	1185	52.7 (49.4–56.1)	0.01
Education			
<High School	3416	65.8 (61.8–69.8)	
High School/Equiv	6148	66.9 (63.6–70.1)	
Some College	7348	65.3 (62.3–68.4)	
College/Grad degree	7090	66.6 (63.6–69.5)	0.66
Insurance Coverage			
Not Covered	4822	65.5 (61.1–69.9)	
Covered	31643	66.2 (64.5–68.0)	0.56
Region			
Northeast	5913	66.1 (61.9–70.4)	
Midwest	7799	66.3 (63.0–69.8)	
South	12887	68.2 (65.5–70.8)	
West	10059	62.6 (59.4–65.8)	0.01

**Table 3:** Relationship between awareness of stroke and calling 911.

N	n (%)	Odds Ratio	OR adjusted	Relative Risk	p
<5 symptoms identified (11554)	10554 (91.7)	1.00 (ref)	1		
All 5 symptoms identified (25104)	24359 (96.9)	2.79(2.42–3.24)	2.64 (2.26–3.08)	1.06 (1.06–1.07)	0.01

OR adjusted for age, sex, race/ethnicity, level of education, insurance coverage, region, hypertension, and diabetes

## Key points

Stroke awareness was demonstrated only in 66% of at risk population in the USA
Among the five stroke symptoms that included sudden weakness of the face or limb, confused/troubled speech, trouble seeing, trouble walking/dizziness/loss of balance; sudden severe headache was the least recognized.
Correct identification of the five stroke symptoms was associated with higher rate of seeking emergency treatment
Racial as well as gender disparity exists regarding stroke symptoms awareness
Further research is needed to help explain the disparity in stroke awareness.
Further efforts and resource allocation should be made to improve stroke awareness particularly among men and minority populations.

## References

[1] National Center for Health Statistics (2014). Mortality multiple cause micro-data files, 2011: public-use data file and documentation: NHLBI tabulations.

[2] (2015). Behavioral Risk Factor Surveillance System: annual survey data, 2013. Centers for Disease Control and Prevention Web site.

[3] Vermeer SE, Longstreth WT, Koudstaal PJ (2007). Silent brain infarcts: a systematic review. Lancet Neurol.

[4] Prabhakaran S, Wright CB, Yoshita M, Delapaz R, Brown T (2008). Prevalence and determinants of subclinical brain infarction: the Northern Manhattan Study. Neurology.

[5] Willey JZ, Williams O, Boden-Albala B (2009). Stroke literacy in Central Harlem: a high-risk stroke population. Neurology.

[6] Kamel H, Navi BB, Fahimi J (2012). National trends in ambulance use by patients with stroke, 1997–2008. JAMA.

[7] Yeo LL, Paliwal P, Teoh HL, Seet RC, Chan BP (2013). Early and continuous neurologic improvements after intravenous thrombolysis are strong predictors of favorable long-term outcomes in acute ischemic stroke. J Stroke Cerebrovasc Dis.

[8] Zerna C, Siepmann T, Barlinn K, Kepplinger J, Pallesen LP (2015). Association of time on outcome after intravenous thrombolysis in the elderly in a telestroke network. J Telemed Telecare.

[R9] 9http://www.healthypeople.gov/2020/topics-objectives/topic/heartdisease-and-stroke/objectives.

[R10] 10http://www.cdc.gov/nchs/nhis/nhis_2014_data_release.htm.

[11] Parsons VL, Moriarity C, Jonas K, Moore TF, Davis KE (2014). Design and Estimation for the National Health Interview Survey, 2006–2015. Vital Health Stat 2.

[12] Howden LM, Meyer JM (2010). Census Briefs. Age and Sex Composition.

[13] Klein RJ, Schoenborn CA (2001). Age adjustment using the 2000 projected U.S. population. Healthy People Statistical Notes.

[14] Eskes GA, Lanctôt KL, Herrmann N (2015). Canadian Stroke Best Practice Recommendations: Mood, Cognition and Fatigue Following Stroke practice guidelines, update 2015. Int J Stroke.

[15] Powers WJ, Derdeyn CP, Biller J, Coffey CS, Hoh BL (2015). AHA/ASA Focused Update of the 2013 Guidelines for the Early Management of Patients With Acute Ischemic Stroke Regarding Endovascular Treatment: A Guideline for Healthcare Professionals From the American Heart Association/American Stroke Association. Stroke.

[16] (2016). Mobile stroke units bring treatment to patients, potentially improving long-term outcomes. ED Manag.

[17] Centers for Disease Control and Prevention (CDC) (2008). Awareness of stroke warning symptoms--13 States and the District of Columbia, 2005. MMWR Morb Mortal Wkly Rep.

[18] Centers for Disease Control and Prevention (CDC) (2004). Awareness of stroke warning signs--17 states and the U.S. Virgin Islands, 2001. MMWR Morb Mortal Wkly Rep.

[19] Center for Disease Control and Prevention (2013). Health Disparities and Inequalities Report-United States. MMWR Morb Mortal Wkly Rep.

[20] Centers for Disease Control and Prevention (CDC) (2005). Disparities in deaths from stroke among persons aged <75 years--United States, 2002. MMWR Morb Mortal Wkly Rep.

[21] Sentell T, Braun K (2012). Low Health Literacy, Limited English Proficiency, and Health Status in Asians, Latinos, and Other Racial/Ethnic Groups in California. J Health Commun.

[22] US Department of Health and Human Services (2000). HP 2010: With Understanding and improving health and objectives for improving health.

[23] Gillum RF, Ingram DD (1996). Relation between residence in the southeast region of the United States and stroke incidence. The NHANES I Epidemiologic Followup Study. Am J Epidemiol.

[24] Lanska DJ (1993). Geographic distribution of stroke mortality in the United States: 1939–1941 to 1979–1981. Neurology.

[25] Casper ML, Wing S, Anda RF, Knowles M, Pollard RA (1995). The shifting stroke belt. Changes in the geographic pattern of stroke mortality in the United States, 1962 to 1988. Stroke.

[26] Glymour MM, Kosheleva A, Boden-Albala B (2009). Birth and adult residence in the Stroke Belt independently predict stroke mortality. Neurology.

[27] Perry HM, Roccella EJ (1998). Conference report on stroke mortality in the southeastern United States. Hypertension.

